# The AERO System: A 3D-Like Approach for Recording Gene Expression Patterns in the Whole Mouse Embryo

**DOI:** 10.1371/journal.pone.0075754

**Published:** 2013-10-16

**Authors:** Hirohito Shimizu, Atsushi Kubo, Kenta Uchibe, Megumi Hashimoto, Shigetoshi Yokoyama, Shuji Takada, Kazuhiko Mitsuoka, Hiroshi Asahara

**Affiliations:** 1 Department of Systems Biomedicine, National Research Institute for Child Health and Development, Tokyo, Japan; 2 Department of Systems Biomedicine, Tokyo Medical and Dental University, Tokyo, Japan; 3 Department of Oral Rehabilitation and Regenerative Medicine, Okayama University Graduate School of Medicine, Dentistry and Pharmaceutical Sciences, Okayama, Japan; Inserm France

## Abstract

We have recently constructed a web-based database of gene expression in the mouse whole embryo, EMBRYS (http://embrys.jp/embrys/html/MainMenu.html). To allow examination of gene expression patterns to the fullest extent possible, this database provides both photo images and annotation data. However, since embryos develop via an intricate process of morphogenesis, it would be of great value to track embryonic gene expression from a three dimensional perspective. In fact, several methods have been developed to achieve this goal, but highly laborious procedures and specific operational skills are generally required. We utilized a novel microscopic technique that enables the easy capture of rotational, 3D-like images of the whole embryo. In this method, a rotary head equipped with two mirrors that are designed to obtain an image tilted at 45 degrees to the microscope stage captures serial images at 2-degree intervals. By a simple operation, 180 images are automatically collected. These 2D images obtained at multiple angles are then used to reconstruct 3D-like images, termed AERO images. By means of this system, over 800 AERO images of 191 gene expression patterns were captured. These images can be easily rotated on the computer screen using the EMBRYS database so that researchers can view an entire embryo by a virtual viewing on a computer screen in an unbiased or non-predetermined manner. The advantages afforded by this approach make it especially useful for generating data viewed in public databases.

## Introduction

In the advent of the post-genomic era, the mechanisms of gene regulation have become a highly active research area. Genome-wide approaches have revealed gene regulatory networks [1], [2] that are comprised of intricate genetic interactions. However, the mechanism by which such networks exert an effect on the body plan during mammalian embryonic development is still far from being well elucidated. To better understand the gene regulatory network in the developmental context, it is essential to document the embryonic gene expression patterns, which are expected to provide clues to elucidate the gene function as well as gene-gene interactions.

Recently, several groups have conducted large-scale gene expression profiling studies using *in situ* hybridization, leading to the development of publicly available on-line gene expression databases [3]–[7]. Many of these databases are advantageous for the investigation of gene expression in highly complex anatomical structures, such as the central nervous system in the late-stage embryo, neonate or adult.

On the other hand, we have focused on the transcription factors involved in early embryogenesis in order to understand the functioning of the transcriptional network in the basic body plan and organogenesis. Thus, we performed comprehensive spatial mapping of transcription factors in a whole mouse embryo on embryonic day 9.5, 10.5 and 11.5 [8]. These images have been collected in the database named EMBRYS (Embryonic gene expression Database as a Biomedical Research Source; http://embrys.jp/embrys/html/MainMenu.html [8]. However, since embryos undergo dynamic three-dimensional morphogenesis during development, it would be of considerable value to be able to record the patterns of gene expression in 3D.

To this end, we have adopted a rotational imaging system, which allows three-dimensional recording of gene expression in the entire embryo by capturing serial 2D images. This approach, termed the AERO system, is optimal for generating images viewed in publicly available databases because a simple procedure results in a motion picture in which the embryo is laterally rotated 360 degrees. We constructed over 800 AERO images of 191 transcription factors that had been shown to be clearly expressed in our previous study [8]. With the addition of the AERO images of these 191 representative genes, the EMBRYS database has been upgraded from the previous version, which contained only conventional 2D images. This investigation underscores the significance of the EMBRYS database and outlines the features of the AERO system as a newly available technique for recording gene expression patterns in the whole embryo.

## Results, Discussion, and Conclusions

### EMBRYS and AERO Images

To build a framework for obtaining a better understanding of the developmental gene regulatory network, we have performed a comprehensive mapping of gene expression of transcription factors in the mouse embryo. EMBRYS is a web-based database that was designed to accommodate all the expression data obtained from this investigation [8]. As with other publicly available databases, conventional 2D images that were photographed following colorimetric detection of gene expression may be easily retrieved from EMBRYS. Typically, an entire view and a partial view focusing on the maxillofacial region, limb buds and internal organs, were captured to provide a profile of the pattern of gene expression. In addition, the limb bud was photographed at a high magnification, since it has been widely studied as a model of morphogenesis due to its simple polarities along the developmental axes [9], [10]. Moreover, additional photos were taken when gene expression was clearly detected in specific organs or tissues. These sets of visual information on gene expression were annotated manually and are provided at the website. The evaluated regions cover multiple tissues and organs, including brain, craniofacial region, limb buds, heart, liver, somites, tail, genitalia and nervous system.

However, since these annotation data are secondary information generated by third parties utilizing the primary data, it is desirable to have a method of recording embryo images from a three-dimensional perspective in order to directly provide raw data, thus maximizing the amount of information obtained from a single image.

**Figure 1:**
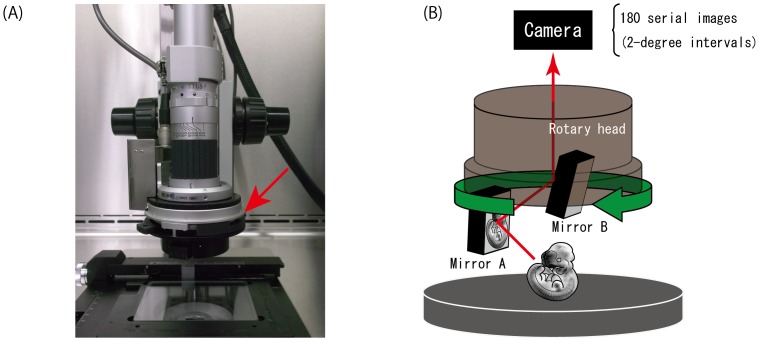
Overall setup of the AERO system hardware. (A) HiRox microscopy and (B) schematic representation of the rotary head. A specimen is placed on an agarose layer prepared in a cylindrical container or petri-dish. The key feature of this component is the two mirrors installed in the rotary head, which allows the camera to capture an image that is tilted 45 degrees to the microscopic stage. The rotary head is rotated by 360 degrees while capturing images at 2-degree intervals. Therefore the 180 serial images are captured in one simple operation. After image acquisition, these serial images are automatically arranged so as to produce a movie.

Therefore, we have enhanced the EMBRYS database by introducing an innovative microscopy system (HiRox, Inc, Japan) that enables the capture of 360-degree rotational images of the whole embryo. By using a rotary head equipped with two mirrors (Fig. 1A) designed to obtain an image tilted at 45 degrees to the microscope stage, images may be captured at 2-degree intervals (Fig. 1B). One operation yields 180 images, which are then serially arranged so as to produce a motion picture (Movie S1). Researchers can easily view the rotating “AERO image” at any lateral angle desired using a computer mouse, by clicking and dragging the edge of the screen, and also by moving the mouse circularly. The basic idea underlying this system is to provide an optimal recording system for database construction by maximizing the data obtained from one specimen in a simple procedure. In fact, since a 360-degree view of the specimen is captured, a significant amount of image data can indeed be recorded in a simple operation, in contrast to conventional 2D images, which are snapshots of one aspect deliberately selected by the observer. This feature allows an entire embryo to be viewed from different angles in an unbiased manner. In particular, AERO images enable the assessment of gene expression in regions conventionally unobservable in a typical 2D lateral view, such as the ventral side of the limb buds, the genitalia and the maxillofacial region.

### Details of the AERO system

Among the 1520 genes analyzed in the previous study [8], we selected 191 genes for which the expression patterns are tissue- or organ-specific for further analysis with the AERO system (Table 1). The expression patterns of the analyzed genes are summarized in Table 2 and Table S1. The captured AERO images were added to the EMBRYS database. In this process, following *in situ* hybridization, the AERO images are captured from both the left and right sides during three developmental stages (E9.5, E10.5 and E11.5). To obtain characteristic gene expression patterns in the craniofacial region and the central nervous system, AERO images were taken from dorsal and ventral sides. (Table 1).

**Table 1:** Genes analyzed in this study.

	Gene Name	No.
Left + Right	*Alx4, Atf1, Atoh1, Barhl1, Barx1, Carm1, Cbfa2t1h, Cdk4, Cdx2, Cebpa, Csda, Dlx3, Dlx4, Dlx5, E130309B19Rik, Ebf1, En1, Eomes, Epas1, Etv5, Evx2, Eya2, Fhl2, Fli1, Foxb1, Foxc2, Foxd1, Foxp4, Fus, Fusip1, Gata1, Gata2, Gata6, Gbx2, Gli1, Gli2, Gli3, Gsc, Hand1, Hand2, Hes1, Hes3, Hes5, Hey1, Heyl, Hmgcs1, Hnrpab, Hoxa10, Hoxa11, Hoxa13, Hoxa7, Hoxa9, Hoxb1, Hoxb13, Hoxb5, Hoxc10, Hoxc5, Hoxc6, Hoxd10, Hoxd12, Hoxd3, Hoxd4, Hoxd9, Hspcb, Idb3, Ing3, Irx1, Irx2, Irx3, Irx5, Isl1, Isl2, Jarid2, Jmjd2a, Khdrbs1, Lbxcor1, Lef1, Lhx1, Lhx2, Lhx8, Lmo2, Lmo4, Mbd2, Meis1, Meox1, Meox2, Mrg1, Msx1, Msx2, Mycn, Myog, Nfatc4, Nhlh2, Nol1, Nr2f2, Nrarp, Onecut1, Otx2, Pax1, Pax3, Pax6, Pax9, Pbx2, Pdlim1, Pdlim4, Peg3, Phf12, Phf21b, Phf5a, Pitx1, Pitx3, Pknox2, Pnrc1, Polr2e, Pou2f2, Pou3f1, Pou3f4, Pou4f2, Prdm1, Prox1, Prrx1, Rqcd1, Runx1, Runx2, Ruvbl1, Ruvbl2, Sall3, Sall4, Scx, Sdccag33, Six1, Smarca4, Smarce1, Smyd1, Snip1, Sox9, Sp6, Sp8, Spdef, Spic, Strap, Tbx15, Tbx18, Tbx2, Tbx3, Tcf7, Tcf7l2, Tead2, Thoc4, Trp63, Tsc22d1, Wwtr1, Ybx1, Zfp219, Zfp238, Zfp385, 1700023B02Rik, 4933406N12Rik*	158
Left + Right + Back + Front	*Aes, Ankrd1, Ankrd15, Arid3a, Bach1, Btg2, Cited1, Cnbp1, Creb3, Ctbp2, Dlx2, Dmrt2, Dmrtc2, EgR1, Elavl2, Ell2, Emx2, En2, Klf6, Lmx1a, Lmx1b, Mcm2, Mllt3, Myf5, Ncor2, Ndn, Ndnl2, Nfia, Nfil3, Nr1h4, Osr2, Taf1, 4932411N23Rik*	33

**Table 2:** The number of genes expressed in respective regions.

	E9.5	E10.5	E11.5
Limb	96 (50.3%)	126 (66.0%)	143 (74.9%)
Somite	75 (39.3%)	95 (49.7%)	96 (50.3%)
Tailbud	25 (13.1%)	29 (15.2%)	23 (12.0%)
Heart	30 (15.7%)	44 (23.0%)	18 (9.4%)
Liver	7 (3.7%)	15 (7.9%)	16 (8.4%)
Maxillary Process	61 (31.9%)	79 (41.4%)	72 (37.7%)
Mandiblar Arch	60 (31.4%)	85 (44.5%)	58 (30.4%)
Hyoid Arch	43 (22.5%)	69 (36.1%)	36 (18.8%)
Eye	37 (19.4%)	55 (28.8%)	64 (33.5%)
External Genitalia	12 (6.3%)	39 (20.4%)	35 (18.3%)
Olfactory Placode	31 (16.2%)	43 (22.5%)	22 (11.5%)
Otic Pit	31 (16.2%)	47 (24.6%)	36 (18.8%)
Brain	56 (29.3%)	66 (34.6%)	67 (35.1%)
Spinal Cord	42 (22.0%)	51 (26.7%)	56 (29.3%)

The population is calculated by dividing by 191, the total number of genes which are analyzed in this study.

**Figure 2:**
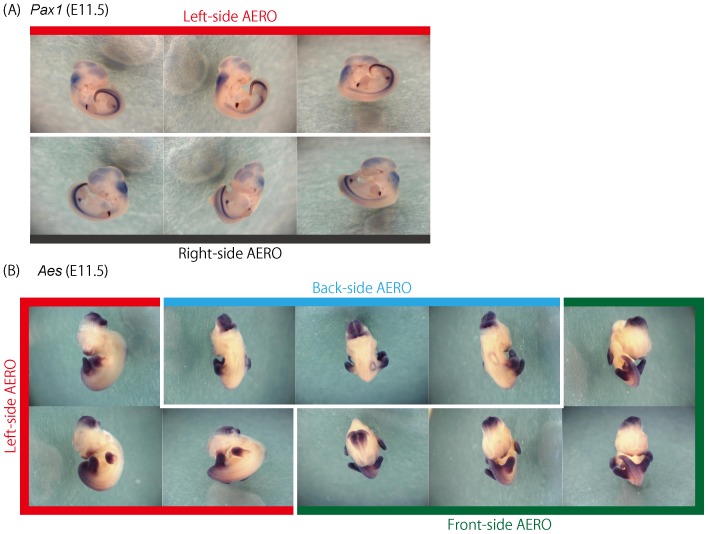
AERO images of *Pax1* and *Aes.* Expression of *Pax1* (paired box gene 1) and *Aes* (amino-terminal enhancer of split) in the E11.5 mouse embryo was visualized by in situ hybridization. (A) Left- and Right-side AERO images of *Pax1*. The expression in the somite and maxillary process is observed. (B)Three AERO images of *Aes*. The Left-, Front- and Back-side AERO images cover almost all the body parts of the embryo. The expression in the brain, somite, forelimb and hindlimb is clearly observed from various angles.

In Figure 2, the Aero images of the *Pax1* and *Aes* genes are shown as an example. *Pax1* is predominantly expressed in somites, pharyngeal arches and limb buds. Aero images of *Pax1*, which cover both the left- and right-side of the embryo, clearly demonstrate the expression of this gene in these regions. Similarly, the AERO images of *Aes* cover the left-, front- and back-side of the embryo, revealing the expression in the hindbrain, limb buds and craniofacial region. Although conventional 2D images also show the expression patterns of each of these genes, its angle is fixed. AERO images of *Pax1* and *Aes* cover multiple angles. For example, in the AERO images of *Pax1* expression at E11.5, the left- and right-side images robustly demonstrate its signals in the entire embryo (Fig. 2A and Movie S2). For *Aes*, the front- and back-side images along with the lateral images provide a comprehensive depiction of the gene expression patterns in many different tissues and organs, such as the brain, craniofacial region, limb buds and tail (Fig. 2B and Movie S3).

### AERO images of *Lmx1b*, *Isl1* and *Cited1*

The AERO system provides a novel means to both obtain and assess gene expression profiles. This is an extremely useful system to apply to databases because a whole embryo can be examined by researchers in an unbiased or unpredetermined manner. In particular, AERO images are useful for the assessment of gene expression in regions not fully observable on a typical 2D lateral view, such as the limb buds, genitalia and maxillofacial region. Here, we focus on three genes, *Lmx1b*, *Isl1* and *Cited1*, as vivid examples in which AERO images provide a viewing advantage.

**Figure 3:**
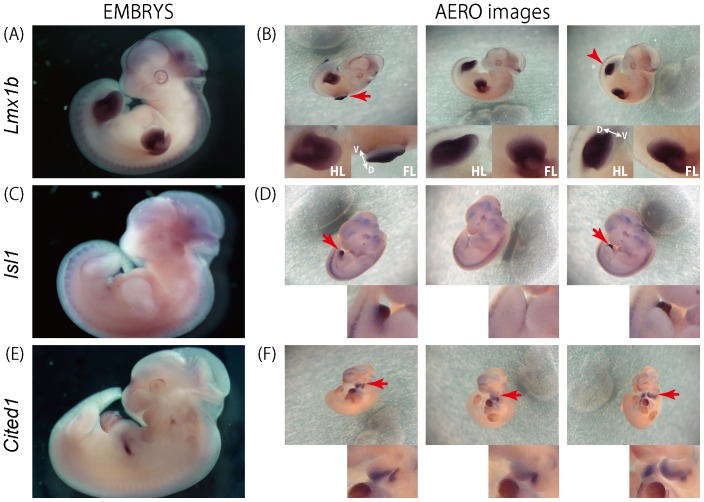
Comparison of conventional 2D images and AERO images. The expression of *Lmx1b*, *Isl1* and *Cited1* at E11.5 is visualized by in situ hybridization. The conventional 2D images (A, C, E) from the EMBRYS database and the AERO images (B, D, F). (A, B) The AERO images reveal *Lmx1b* expression is restricted to the dorsal side and is absent on the ventral side of the forelimb-bud (arrow) and hindlimb bud (arrow head). D:dorsal side, V: ventral side. (C, D) *Isl1* expression in the genitalia is clearly observed in the AERO images (red arrows). (E, F) *Cited1* expression in the maxillofacial region is more clearly observed in the AERO images than 2D image (red arrows).

The *Lmx1b* gene is expressed in the limb buds at E11.5, but its expression in the limb buds is restricted to the dorsal side. In conventional 2D photographs, it is difficult to distinguish the dorsal and ventral side (Fig.3A). However, in the AERO images, the specific expression in the dorsal side is clearly observed in both forelimbs (the arrow) and hindlimbs (the arrow-head) (Fig.3B and Movie S4). In the case of *Isl1*, the expression in the genitalia can not be observed in the conventional 2D images while the AERO images depict the expression in this region (Fig. 3C, D and Movie S5). Similarly, while *Cited1* expression visibility in the craniofacial region is very limited on the 2D view, the observation of gene expression from multiple angles using the AERO system reveals its expression in the medial pharyngeal arches (Fig.3E, F and Movie S6).

### Future directions

The major challenge for the AERO system is to continuously increase the amount of data contained within it while also expanding the database user features. We continue to gather AERO images to cover all of the genes assayed for the EMBRYS project in order to create a more comprehensive gene expression atlas of the whole mouse embryo. We would like to encourage researchers all over the world to utilize this expression information in their own studies and to expand upon it in any that fits with their own expertise.

In addition to the AERO system itself, we will continue to update the whole EMBRYS database by adding still more useful features. Using the comprehensive data of whole-mount gene expression mapping, further description of the gene expression that takes place in complex anatomical structures will be investigated by sectional *in situ* hybridization. In fact, a pilot study has been conducted to identify genes expressed in the tooth germ in our laboratory, using an approach in which genes found to be expressed in the maxillofacial region were selected according to the data in EMBRYS [11]. Furthermore, we have started communicating with other gene expression databases for the purpose of collaborative interaction. As the initial step, we included a direct link in each gene expression profile page to corresponding entries at EMAGE and MGI so as to allow users to more precisely evaluate the gene expression by viewing a variety of images.

AERO images can be easily obtained by a less laborious procedure than other complicated methods, which is basically the same as that used to obtain conventional 2D images; an assayed embryo is placed under the microscope and serial images are captured automatically after being manually focused. For this procedure, no particular treatment or processing is required, unlike other 3D imaging systems such as 3D reconstruction of tissue sections [12], [13] and optical projection tomography (OPT) [14], [15]. Thus, all the images are provided as primary data that are directly obtained from the embryo by a non-destructive method and the images are highly reproducible regardless of individual expertise. These features are advantageous to a large-scale assay in which numerous images are produced.

## Materials and Methods

### Ethics statement

All animal experiments were performed according to protocols approved by the Institutional Animal Care and Use Committee at National Institute for Child Health and Development (Protocol 2004-003).

### Preparation of Embryos

Pregnant ICR mice were purchased from Sankyo Labo Service Cooperation Inc. (Tokyo, Japan). At E9.5, 10.5 and 11.5, embryos were manually harvested into RNase-free cold PBS treated with DEPC and fixed overnight in a solution of 4% paraformaldehyde in PBS at 4°C. Embryos were subsequently dehydrated through a graded series of methanol washes (25%, 50%, 75% and twice with 100%) in PBT (PBS containing 0.1% Tween-20) and stored at –20°C until needed for WISH assays.

### Whole-Mount *In Situ* Hybridization

Prior to probe synthesis, the coding region in each gene was amplified from the mouse cDNA library [16] by add-on mutagenesis PCR, so that the Sp6 RNA polymerase promoter site was added at the 5’ end of the PCR product. Following amplification, cRNA riboprobes were synthesized with Sp6 RNA polymerase according to the standard procedure in the presence of digoxigenin-conjugated UTP. A 96-well plate was used for the whole procedure in order to produce 96 probes simultaneously in each reaction.

Whole mount *in situ* hybridization (WISH) assays were performed based on the standard protocol. However, to assay many genes efficiently, some modifications introduced for embryo handling. For a detailed description of both the probe preparation and WISH assays, see Shimizu *et al* [17].

### The Capture and Reconstitution of Images

2D images were serially captured from multiple angles by HiRox microscopy (KH-1300, HiRox, Inc, Japan) as follows. An embryo was place on agarose gel prepared in a cylindrical container so that it was stably positioned in various orientations. The rotary head equipped with ring lighting was connected to the microscope and rotational images were captured at 2-degree intervals using a CCD camera. The images were saved in the “AERO” format (an executable file), which contains both the picture data and the viewer program. Users can inspect each expression without the need of any additional software.

The raw data and AERO images are available for download from the EMBRYS website (http://embrys.jp/embrys/html/MainMenu.html).

## Supporting Information

Table S1 **Expression patterns of the analyzed genes.** The expression patterns of 191 genes are annotated.
(XLSX)

Movie S1 **AERO image of** ***Sox9.*** The AERO image of *Sox9* is shown as an example. 180 images which were captured by the rotary head are serially arranged to produce a motion picture.
(WMV)

Movie S2 **AERO image of** ***Pax1.*** In the AERO images of Pax1 expression at E11.5, the left- and right-side images robustly demonstrate its signals in the entire embryo.
(WMV)

Movie S3 **AERO image of** ***Aes.*** In the AERO images of *Aes*, the front- and back-side images along with the lateral images provide a comprehensive depiction of the gene expression patterns in many different tissues and organs.
(WMV)

Movie S4 **AERO image of** ***Lmx1b.*** In the AERO image of *Lmx1b* at E11.5 embryo, the specific expression in the dorsal side is clearly observed in both forelimbs and hindlimbs.
(WMV)

Movie S5 **AERO image of** ***Isl1.*** The AERO image of *Isl1* at E11.5 embryo clearly depicts the expression in the genitalia.
(WMV)

Movie S6 **AERO image of** ***Cited1.*** The AERO image of *Cited1* at E11.5 embryo reveals its expression in the medial pharyngeal arches.
(WMV)
